# National and subnational estimation of the prevalence of peripheral artery disease (PAD) in China: a systematic review and meta-analysis

**DOI:** 10.7189/jogh.09.010601

**Published:** 2019-06

**Authors:** Peige Song, Diana Rudan, Manli Wang, Xinlei Chang, Igor Rudan

**Affiliations:** 1 Centre for Global Health Research, Usher Institute of Population Health Sciences and Informatics, University of Edinburgh, Edinburgh, Scotland, UK; 2 Clinical Hospital Dubrava, Zagreb, Croatia; 3 Department of Maternal and Child Health, School of Public Health, Peking University, Beijing, China

## Abstract

**Background:**

Peripheral artery disease (PAD), the third leading cause of atherosclerotic vascular morbidity, affects approximately 202 million people worldwide, among whom more than two-thirds reside in low- and middle-income countries (LMIC). For China, the largest developing country, little is known about the epidemiology of PAD. We aimed to estimate the prevalence of PAD and the number of affected people in China, establish the main risk factors for PAD and assess the number of people with PAD at the sub-national level.

**Methods:**

We searched China National Knowledge Infrastructure (CNKI), Wanfang, Chinese Biomedicine Literature Database (CBM-SinoMed), PubMed, Embase and Medline for population-based studies that have reported the prevalence of PAD in the general Chinese population from 1990 onwards. PAD was defined as an ankle-brachial index (ABI) lower than or equal to 0.90. We used a multilevel mixed-effects logistic regression to generate the gender- and age-specific prevalence of PAD, and a random-effects meta-analysis to pool the odds ratios (ORs) of major risk factors. United Nations population numbers were used to estimate and project the number of affected people from 2000 to 2020. Finally, we used the risk factors-based model to distribute the national number of people with PAD into different settings (urban and rural) and regions (East, Central and West) for the year 2010.

**Results:**

Overall, 37 articles met all inclusion criteria and provided prevalence estimates, among which 14 also explored risk factors for PAD. The prevalence of PAD increased gradually by age until mid-60s, after which the increase accelerated. In males, the prevalence of PAD ranged from 2.81% (95% CI = 1.77-4.43) in those aged 25-29 years to 21.95% (95% CI = 15.39-30.31) in those 95-99 years old. In females, the PAD prevalence increased from 3.84% (95% CI = 2.44-5.98) in those aged 25-29 years to 27.95% (95% CI = 20.14-37.37) in those aged 95-99 years. The PAD prevalence was consistently higher in females than in males across all age groups. This difference was most significantly pronounced among the elderly, starting from 60 years. Between 2000 and 2020, the total number of Chinese people with PAD is expected to increase by 40%: from 29.44 million (95% CI = 22.51-38.62) in 2000 to 41.13 million (95% CI = 32.00-52.95) in 2020. Current smoking was the strongest risk factor for PAD, with a meta-odds ratio (OR) of 2.62 (95% CI = 1.44-4.76), followed by hypertension (1.94, 95% CI = 1.48-2.53) and diabetes (1.71, 95% CI = 1.45-2.01). In 2010, 15.18 million (95% CI = 11.74-19.67) people with PAD resided in the East China, 11.08 million (95% CI = 8.61-14.28) in the Central China and 8.65 million (95% CI = 6.71-11.16) in the West China. In addition, 24.20 million (95% CI = 18.82-31.16) people with PAD were living in rural areas, accounting for almost 70% of all PAD cases in China.

**Conclusions:**

With rapid ageing in China, PAD has become a serious public health problem. More research and optimal interventions on PAD are required to better identify effective strategies for prevention and treatment of PAD in China.

Peripheral artery disease (PAD), atherosclerotic obstruction of the arteries of the lower extremities, involves a wide range of disorders of the structure (including stenosis and aneurysms) and function of all non-coronary arteries [1]. The classic manifestations of PAD include intermittent claudication, atypical leg pain, acute limb ischemia and critical limb ischemia, along with diminished productivity and quality of life [2,3]. Even regardless of symptoms, patients with asymptomatic PAD are associated with an increased risk of incident cardiovascular events morbidity and mortality, placing a large burden on caregivers and community [4-6]. According to the global PAD study, PAD, as measured by the abnormal ankle-brachial index (ABI, the ratio of the systolic blood pressure at the ankle to the systolic blood pressure in the arm) seems to affect roughly 202 million people in the world, more than two thirds of whom residing in low- and middle-income countries (LMIC) [7]. Between 2000 and 2010, the number of affected people worldwide has increased by 23.51%, where the increasing rate in LMIC was twice higher than that in high-income countries (HIC) (28.67% vs 13.08%) [7]. As a widespread disorder, PAD is particularly common in elderly and patients with cardiovascular risk factors [1,4,7-9]. As the global population continues to age and the burden of noncommunicable diseases (NCDs) keeps rising, it seems that PAD will subsequently pose a substantial challenge to health care systems and the whole society in the future [1,10].

PAD is the third leading cause of atherosclerotic vascular morbidity, after coronary heart disease and stroke. Its morbidity, mortality and health care costs are comparable to, or even higher, than for the other two diseases [7,11-13]. Unfortunately, compared with abundant studies on coronary heart disease and stroke, PAD is still relatively neglected in terms of research attention. This is in spite of its magnitude, morbidity and mortality implications [4,11,14,15]. The first estimate of the worldwide burden of PAD has raised a call for further epidemiological studies and more actions in secondary prevention of PAD [7,16,17]. To date, synthesised estimation of PAD prevalence is still largely hindered because of the paucity of epidemiological data, especially in LMIC [7,16,18]. The unavoidable over-reliance on studies from HIC may twist the overall global perspective of PAD burden and its distributions. Further epidemiological studies in LMIC are therefore much needed [7,16]. In addition, previous evidence has suggested marked variations in the epidemiology of PAD and its risk factors. For example, it was reported that the prevalence of PAD did not differ meaningfully between genders, whereas the predominance of women with PAD was seen in many studies in LMIC [7,8,16,19]. The regional, ethnic and gender heterogeneity highlights the need for in-depth evidence-based initiatives to generalise the burden of PAD at local levels and on a regular basis [11,20-22].

In the largest LMIC country, China, the past several decades of fair economic progress have seen a substantial rise in the burden of NCDs. This rise has mainly been driven by an ageing population and unfavourable increasing trends for relevant risk factors [23-25]. Without adequate measures to combat this emerging trend of NCDs, the burden of PAD may also become a special challenge for the Chinese health care system. Under this circumstance, an insight into the prevalence of PAD and its risk factors in general Chinese population is essential for the management of PAD patients and guiding secondary preventive measures, and therefore of substantial interest to both policymakers and academics [16]. The growing body of data in China's bibliographic databases provides a good opportunity to explore the prevalence of PAD in China [26-28]. Until recently, no studies have systematically examined the burden of PAD in China. To fill this knowledge gap, we conducted a systematic review of existing literature in both English and Chinese bibliographic databases to estimate the first age- and gender-specific prevalence of PAD in China. We also aimed to explore the main risk factors for PAD and to provide estimates of the number of people living with PAD at different settings (urban vs rural) and geographic regions.

## METHODS

### Search strategy and study eligibility

Two researchers (MW and XC) independently performed a comprehensive systematic review of published literature that reported the epidemiology of PAD in China, following the Preferred Reporting Items for Systematic reviews and Meta-Analysis (PRISMA) guidelines and the Guidelines for Accurate and Transparent Health Estimates Reporting (GATHER) standards [29,30]. The searched bibliographic databases included three English databases (Medline, PubMed and Embase) and three Chinese databases (China National Knowledge Infrastructure [CNKI], Wanfang data and Chinese Biomedicine Literature Database [CBM-SinoMed]). As listed in Table S1 in **Online Supplementary Document**, specific search strategies were developed to adapt different searching characteristics of each database. The search terms were combinations of “incidence” or “prevalence” or “epidemiology” or “morbidity” or “mortality”, and “peripheral artery disease” and “China or Chinese”, in the forms of free words or controlled vocabulary (ie, medical subject headings). In addition, snowball searching was conducted to locate any potentially relevant studies from the reference lists of all full-text articles. No language restrictions were applied.

Only studies published from January 1990 onwards were included in the systematic review. Articles were independently examined in two stages: screening of titles, abstracts and keywords and the selection of full-text articles. Studies were eligible for inclusion if they met the following eligibility criteria: population-based studies that reported the epidemiology of PAD in general Chinese population; to be in line with the Global PAD study, eligible studies should have defined PAD as an ABI value lower than 0.90 or equal to or lower than 0.90 [7,16,31]. Studies that were conducted in special populations (eg, diabetic patients, inpatients) were deemed as not to be representative of the general population, and hence were not included in our analysis. We also excluded studies that only adopted qualitative methods. For multiple publications reported on the same single study, the one providing the most extensive information or the most recent one was kept for further analysis. A total of 37 studies met the eligibility criteria.

### Data extraction

Three researchers (PS, MW and XC) independently extracted data from included studies, using a purpose-built form. The following data items were abstracted, when provided: author, title, year of publication, geographic location, years of investigation, study design, sampling method, diagnostic method and outcome definition, age range and reported prevalence of PAD (the number of participants and PAD cases). For studies without a specific year of investigation, three years were subtracted from their year of publication, based on the average difference of the year of investigation and the publication year where data were available (see Table S2 in **Online Supplementary Document**). If prevalence estimate were reported by age, gender or setting (urban and rural) within the same study, information was split into each subgroup separately. For studies that reported the risk factors for PAD, estimates of odds ratios (ORs) and corresponding confidence intervals (CIs) based on multivariate study design were also extracted. There were 14 such studies. All disagreements at both the screening and data extraction stages were resolved by consensus.

### Data synthesis procedures and statistical analyses

The data synthesis procedures are in line with those in our previous global PAD study [7].

#### Epidemiological modelling of age- and gender-specific prevalence of PAD

In data extraction stage, multiple outcome data points were extracted from the same individual study. Finally, a total of 166 data points from 37 studies contributed to the overall database. The extracted prevalence estimates of PAD were re-calculated from the raw proportions and stabilised by the logit transformation [32]. To take into account the occurrence of repeated sample from the same study, a multilevel mixed-effects logistic regression approach was adopted [7,33]. First, univariable meta-regression was adopted to explore the association of prevalence estimates and each individual factor, ie, year of investigation, age, gender (male vs female) and setting (urban vs rural). Any factors that were significantly associated with the prevalence estimates of PAD in the univariable meta-regression were subsequently added into the multivariable meta-regression analysis (see Table S3 in **Online Supplementary Document** for more details). The final model included age and gender, where the nonlinear relation of age and PAD prevalence was modelled by using restricted cubic splines [34,35]. Given that: Prevalence = p = PAD cases / number of participants,

Then, the prevalence estimates were stabilized by using the logit link,


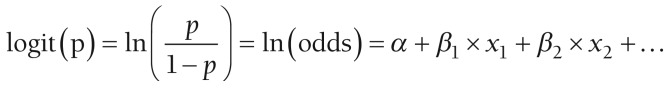


As gender and age (in the form of restricted cubic splines: Age_1_ – Age_4_) are the covariates included in the final model,

Logit (p) =α +β × Gender + β_1_ × Age_1_ + β_2_ × Age_2_ + β_3_ × Age_3_ + β_4_ × Age_4_

Thus,


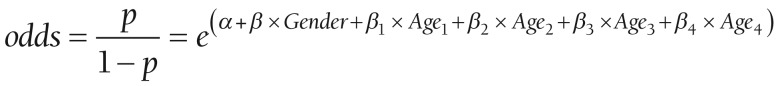


And,


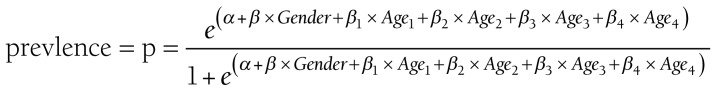


Finally, age- and gender-specific prevalence of PAD was generated based on the above-mentioned model.

#### Estimation of the national number of PAD cases in the years 2000, 2010 and the projection for 2020

By multiplying the age- and gender-specific prevalence with the corresponding Chinese population, available from the United Nations Population Division (UNPD), the numbers of people living with PAD across all age groups were calculated for the years 2000, 2010 and 2020, respectively [36]. This was done for every 5-year age group, ranging from 25-29 years to 95-99 years. Then, the overall number of people living with PAD in the years 2000, 2010 and 2020 was derived by adding the age- and gender-specific cases together.

#### Meta-analyses of risk factors

A total of 76 data points from 14 studies provided OR estimates and CIs for risk factors. These data points were then clustered into different risk factor groups based on their definitions. As a rule, we only included the factors where at least three independent studies were available [7]. Finally, nine risk factors (age, gender, current smoking, systolic blood pressure [SBP], diastolic blood pressure [DBP], current drinking, body mass index [BMI], hypertension and diabetes) were included for subsequent meta-analyses by using a random-effects (DerSimonian and Laird method) model.

#### Estimation of subnational PAD cases in 2010

At the final stage, the national number of people with PAD in 2010 was distributed into different regions by combining the pooled ORs generated at stage 3 with subnational prevalence of major risk factors, this was done on the basis of the difference in the prevalence of major risk factors between urban and rural settings and in different regions (East, Central and West) in China [7,37-39]. Three risk factors (current smoking, hypertension and diabetes) were chosen because they were significantly associated with the prevalence of PAD and had corresponding subnational prevalence data. We obtained the prevalence of these risk factors from the 2010 China Non-communicable and Chronic Disease surveillance [39], whose definitions of these three risk factors were in line with those extracted from included articles (Table S4 in **Online Supplementary Document**).

All statistical analyses were performed using R version 3.3.0 (R Foundation for Statistical Computing, Vienna, Austria) and STATA version 14.0 (STATA Corporation, College Station, TX, USA). The China map was drawn by ArcMap version 10.1 (Environmental Systems Research Institute, Redlands, CA). Statistical tests were two-sided and a *P* < 0.05 indicated statistical significance.

## RESULTS

### Identification and characteristics of studies

**Figure 1:**
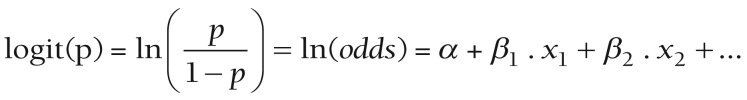
PRISMA study selection flow diagram.

Our initial search yielded a total of 7828 records. After removing 2102 duplicates, 5726 unique records were screened for relevance by titles and abstracts. Then, we reviewed the full text of 354 articles. Finally, 37 articles met inclusion criteria and provided prevalence estimates, among which 14 explored risk factors for PAD (**Figure 1**).

**Figure 2:**
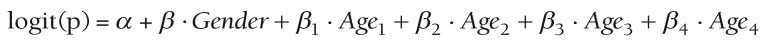
Geographic distribution of the included studies. The classification of East, Central and West was based on the 31 provinces’ geography and economic development [38,39]. The most developed region (East) includes Beijing, Tianjin, Hebei, Liaoning, Shanghai, Jiangsu, Zhejiang, Fujian, Shandong, Guangdong and Hainan; The least developed region (West) includes Inner Mongolia, Chongqing, Guangxi, Sichuan, Guizhou, Yunnan, Tibet, Shaanxi, Gansu, Qinghai, Ningxia, and Xinjiang; The Central region includes Shanxi, Jilin, Heilongjiang, Anhui, Jiangxi, Henan, Hubei and Hunan.

All the included studies were cross-sectional investigations. Table S5 in **Online Supplementary Document** shows the detailed characteristics of each study. Briefly, the 37 studies included a total of 132 368 participants (6888 with PAD) and provided 166 data points of PAD prevalence estimates. Years of publication ranged from 2005 to 2016. 18 studies were conducted in urban areas, 12 in rural areas and seven in mixed areas. The geographic distribution of the included studies is shown in **Figure 2**.

### Gender- and age-specific prevalence of PAD in China

**Figure 3:**
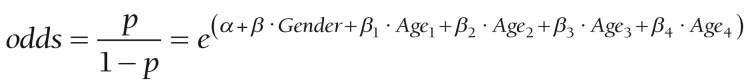
Gender- and age-specific prevalence of peripheral artery disease (PAD) based on the informative data points from the included studies The size of each bubble is proportional to sample size.

Based on informative data points from the included studies, the relation between age and prevalence of PAD in both males and female was developed (**Figure 3**). There were substantial numbers of data points across most of the age spectrum. Generally, the prevalence of PAD increased steadily, but slowly, with advanced age from early 30s to late 60s. Then, it started to increase dramatically after the age of 70 years.

As shown in the Table S3 in **Online Supplementary Document**, only age and gender were significantly associated with the prevalence of PAD. The final model generated from the multilevel mixed-effects logistic regression is given below:

ln(odds) = 3.088-0.322 × *Gender_Male_* + 0.007 × Age_1_ –0.031 × Age_2_ + 0.139 × Age_3_ – 0.122 × Age_4_

Where:

odds = p / (1 – p), *p* is the prevalence of PAD,

Gender_Male_ = 1 for males and 0 for females

Age_1_ to Age_4_ represent variables derived from the process of fitting restricted cubic splines (knots: 31.58, 45.41, 64.50, 74.00, 84.50).

**Figure 4:**
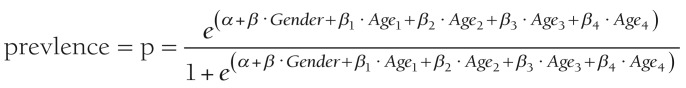
Estimated gender- and age-specific prevalence of peripheral artery disease (PAD)in China, with 95% confidence interval (CI).

The gender- and age-specific prevalence of PAD in China was calculated based on the final model (**Table 1** and **Figure 4**). In males, the prevalence of PAD ranged from 2.81% (95% CI = 1.77-4.43) in young adults aged 25-29 years to 21.95% (95% CI = 15.39-30.31) in those were 95-99 years old, which represented an almost 10-fold increase. The prevalence of PAD increased slowly until middle-60s when it increased exponentially. The trend of PAD prevalence by age was similar between genders. In females, the PAD prevalence increased from 3.84% (95% CI = 2.44-5.98) in those aged 25-29 years to 27.95% (95% CI = 20.14-37.37) in those aged 95-99 years. The PAD prevalence was consistently higher in female than in males across all age groups, and this difference was most significantly pronounced in elderly, starting from 60 years.

**Table 1:** Gender-specific prevalence of PAD in China, by age group

Age group (years)	Prevalence of PAD (%, 95% CI)	
**Male**	**Female**
25-29	2.81 (1.77-4.43)	3.84 (2.44-5.98)
30-34	2.91 (2.09-4.03)	3.97 (2.88-5.43)
35-39	3.00 (2.35-3.83)	4.09 (3.25-5.14)
40-44	3.07 (2.43-3.88)	4.19 (3.37-5.20)
45-49	3.09 (2.42-3.95)	4.22 (3.36-5.29)
50-54	3.08 (2.44-3.89)	4.20 (3.38-5.22)
55-59	3.12 (2.49-3.89)	4.25 (3.45-5.22)
60-64	3.29 (2.64-4.09)	4.48 (3.65-5.50)
65-69	3.73 (3.00-4.61)	5.07 (4.14-6.18)
70-74	4.58 (3.66-5.70)	6.20 (5.04-7.61)
75-79	6.05 (4.83-7.56)	8.16 (6.62-10.03)
80-84	8.40 (6.71-10.47)	11.23 (9.12-13.75)
85-89	11.75 (9.13-15.01)	15.52 (12.27-19.44)
90-94	16.21 (11.99-21.56)	21.07 (15.91-27.35)
95-99	21.95 (15.39-30.31)	27.95 (20.14-37.37)

PAD – peripheral artery disease, CI – confidence interval

### National number of people with PAD in the years 2000, 2010 and 2020

By applying the estimated gender- and age-specific prevalence of PAD to the national demographic data, the number of people living with PAD in China was calculated for the years 2000, 2010 and 2020 respectively (**Table 2**). During the two decades, the total number of Chinese people living with PAD is expected to increase by 39.71% - from 29.44 million (95% CI = 22.51-38.62) in 2000 to 41.13 million (95% CI = 32.00-52.95) in 2020. The age groups with significant increases will be 50-59 years, and 65-69 years, as well as above 85 years, where rates of change in the number of PAD cases are expected to all be above 100%. However, in younger people aged under 29 years and those aged 35-39 years, the number of people living with PAD will decrease throughout the same period. Moreover, the increasing rate in females will be higher than that in males from 40 years to 74 years, and then it will become lower from 75 years onwards. By 2020, 57.50% of the PAD cases in China will be females.

**Table 2:** Estimated number of people living with PAD in China in the years 2000, 2010 and 2020, and the rate of change from 2000 to 2020, by gender and age group

Age group (years)	People living with PAD in 2000 (million, 95% CI)			People living with PAD in 2010 (million, 95% CI)			People living with PAD in 2020 (million, 95% CI)			Rate of change (%, 2000-2020)		
**Male**	**Female**	**Overall**	**Male**	**Female**	**Overall**	**Male**	**Female**	**Overall**	**Male**	**Female**	**Overall**
25-29	1.75	2.26	4.02	1.48	1.92	3.40	1.49	1.83	3.32	-14.86	-19.03	-17.41
	(1.10-2.76)	(1.44-3.53)	(2.54-6.29)	(0.94-2.34)	(1.22-2.99)	(2.15-5.33)	(0.94-2.35)	(1.16-2.85)	(2.10-5.20)			
30-34	1.89	2.49	4.38	1.44	1.87	3.31	1.94	2.47	4.41	+2.65	-0.80	+0.68
	(1.36-2.63)	(1.81-3.41)	(3.16-6.03)	(1.04-2.00)	(1.36-2.56)	(2.39-4.56)	(1.39-2.69)	(1.80-3.39)	(3.19-6.08)			
35-39	1.63	2.12	3.75	1.84	2.39	4.23	1.56	2.02	3.59	-4.29	-4.72	-4.27
	(1.27-2.08)	(1.68-2.66)	(2.95-4.74)	(1.44-2.35)	(1.89-3.00)	(3.33-5.35)	(1.22-2.00)	(1.60-2.54)	(2.83-4.54)			
40-44	1.33	1.72	3.06	1.96	2.59	4.55	1.50	1.95	3.45	+12.78	+13.37	+12.75
	(1.05-1.68)	(1.39-2.14)	(2.44-3.82)	(1.55-2.48)	(2.08-3.21)	(3.63-5.69)	(1.19-1.90)	(1.57-2.42)	(2.75-4.31)			
45-49	1.38	1.79	3.17	1.64	2.14	3.78	1.86	2.42	4.28	+34.78	+35.20	+35.02
	(1.08-1.76)	(1.43-2.25)	(2.50-4.01)	(1.28-2.09)	(1.70-2.69)	(2.99-4.78)	(1.46-2.37)	(1.93-3.04)	(3.38-5.41)			
50-54	0.97	1.26	2.23	1.29	1.69	2.98	1.91	2.54	4.46	+96.91	+101.59	+100.00
	(0.76-1.22)	(1.01-1.57)	(1.78-2.79)	(1.02-1.63)	(1.36-2.09)	(2.38-3.72)	(1.51-2.41)	(2.04-3.16)	(3.56-5.57)			
55-59	0.75	0.94	1.70	1.32	1.74	3.06	1.58	2.09	3.67	+110.67	+122.34	+115.88
	(0.60-0.94)	(0.77-1.16)	(1.37-2.10)	(1.05-1.64)	(1.41-2.13)	(2.47-3.78)	(1.26-1.97)	(1.70-2.56)	(2.96-4.53)			
60-64	0.72	0.89	1.61	0.95	1.26	2.21	1.27	1.70	2.98	+76.39	+91.01	+85.09
	(0.57-0.89)	(0.73-1.09)	(1.30-1.98)	(0.76-1.18)	(1.03-1.55)	(1.79-2.72)	(1.02-1.59)	(1.38-2.09)	(2.41-3.67)			
65-69	0.67	0.87	1.54	0.77	1.00	1.77	1.36	1.87	3.24	+102.99	+114.94	+110.39
	(0.54-0.82)	(0.71-1.07)	(1.25-1.89)	(0.62-0.95)	(0.82-1.22)	(1.44-2.18)	(1.10-1.69)	(1.53-2.29)	(2.63-3.98)			
70-74	0.56	0.78	1.35	0.75	1.00	1.75	1.01	1.45	2.46	+80.36	+85.90	+82.22
	(0.45-0.70)	(0.64-0.96)	(1.09-1.66)	(0.60-0.93)	(0.82-1.23)	(1.41-2.16)	(0.81-1.26)	(1.18-1.78)	(1.99-3.04)			
75-79	0.43	0.70	1.13	0.64	0.97	1.61	0.78	1.16	1.93	+81.40	+65.71	+70.80
	(0.34-0.53)	(0.57-0.86)	(0.91-1.39)	(0.51-0.80)	(0.79-1.20)	(1.30-2.00)	(0.62-0.97)	(0.94-1.42)	(1.56-2.39)			
80-84	0.31	0.58	0.89	0.44	0.75	1.19	0.64	1.02	1.66	+106.45	+75.86	+86.52
	(0.25-0.39)	(0.47-0.71)	(0.72-1.09)	(0.35-0.55)	(0.61-0.92)	(0.96-1.47)	(0.51-0.79)	(0.83-1.25)	(1.34-2.04)			
85-89	0.15	0.32	0.47	0.23	0.46	0.70	0.39	0.71	1.10	+160.00	+121.88	+134.04
	(0.11-0.19)	(0.25-0.40)	(0.37-0.59)	(0.18-0.30)	(0.37-0.58)	(0.55-0.88)	(0.30-0.50)	(0.56-0.89)	(0.86-1.39)			
≥90	0.04	0.13	0.18	0.11	0.26	0.37	0.19	0.41	0.60	+375.00	+215.38	+233.33
	(0.03-0.06)	(0.10-0.17)	(0.13-0.23)	(0.08-0.15)	(0.20-0.34)	(0.28-0.49)	(0.14-0.25)	(0.31-0.54)	(0.44-0.79)			
Total	12.57	16.87	29.44	14.86	20.05	34.90	17.49	23.65	41.13	+39.14	+40.19	+39.71
	(9.53-16.65)	(12.99-21.97)	(22.51-38.62)	(11.42-19.39)	(15.65-25.72)	(27.07-45.10)	(13.47-22.74)	(18.53-30.21)	(32.00-52.95)			

CI – confidence interval

### Synthesized effect size of risk factors for PAD

A total of 14 studies provided data on risk factors for PAD by using multivariate design. After clustering all the risk factors by their specific definitions, nine risk factors had sufficient information and were therefore included for synthesis (**Table 3**). Female gender was found to be a significant risk factor for PAD, this was in line with our estimates on the gender-specific prevalence of PAD in China. In addition, current smoking, hypertension and diabetes also showed significant associations with PAD. Current smoking was the strongest risk factor, with a meta-OR of 2.62 (95% CI = 1.44-4.76), followed by hypertension (1.94, 95% CI = 1.48-2.53) and diabetes (1.71, 95% CI = 1.45-2.01). The detailed meta-analysis process and information of individual studies can be found in Table S6 in **Online Supplementary Document**.

**Table 3:** Synthesized effect size of 9 risk factors that were investigated in at least three studies using multivariate design

Risk factor	Number of studies	OR (95% CI)	z value	*P*-value
Risk factor 1-Advanced age (per year)	8	1.01 (0.98-1.03)	0.37	0.715
Risk factor 2-Female gender	10	1.61 (1.08-2.40)	2.35	0.019
Risk factor 3-Current smoking	5	2.62 (1.44-4.76)	3.17	0.002
Risk factor 4-SBP (mmHg)	4	1.01 (0.96-1.05)	0.24	0.809
Risk factor 5-DBP (mmHg)	3	1.02 (0.96-1.08)	0.61	0.54
Risk factor 6-Current drinking	3	1.15 (0.93-1.43)	1.33	0.184
Risk factor 7-BMI (kg/m^2^)	4	0.92 (0.84-1.02)	1.59	0.111
Risk factor 8-Hypertension	6	1.94 (1.48-2.53)	4.86	<0.001
Risk factor 9-Diabetes	7	1.71 (1.45-2.01)	6.49	<0.001

OR – odds ratio, CI – confidence interval, SBP – systolic blood pressure, DBP – diastolic blood pressure, BMI – body mass index

### Subnational number of people with PAD in 2010

**Figure 5:**
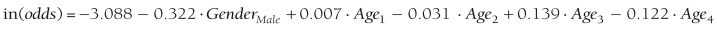
Estimated number of people living with peripheral artery disease (PAD)and contributing age groups in the three regions of China, 2010.

According to variations of the prevalence of the three major risk factors: current smoking, hypertension and diabetes (**Table S4** in **Online Supplementary Document**) across China, the national number of PAD cases in 2010 was distributed to three regions and split into different settings (urban and rural). **Table 4** and **Figure 5** illustrate that in 2010, 15.18 million (95% CI = 11.74-19.67) people with PAD resided in the East China, 11.08 million (95% CI = 8.61-14.28) in the Central China and 8.65 million (95% CI = 6.71-11.16) in the West China. The distribution of PAD cases between urban and rural settings was also uneven in all the three regions, with the majority (69.32%, 24.20 million [95% CI = 18.82-31.16]) living in rural areas. In rural areas of the three regions, the age group that contributed most PAD cases was 40-44 years. Most PAD cases in urban East China were aged 25-29 years, whereas most PAD cases were noted in people aged 35-39 years in urban West China and in people aged 40-44 years in urban Central China. This finding highlights the younger demographic structure of the urban setting than the rural setting, especially in East China.

**Table 4:** Estimated number of people living with PAD in China in 2010, by region, setting and age group

Age group	People living with PAD in 2010 (million, 95% CI)								
**Urban**			**Rural**			**Overall**		
**Central**	**East**	**West**	**Central**	**East**	**West**	**Central**	**East**	**West**
25-29	0.26	0.80	0.21	0.73	0.81	0.58	0.99	1.62	0.80
	(0.17-0.41)	(0.51-1.26)	(0.14-0.33)	(0.46-1.14)	(0.52-1.28)	(0.37-0.91)	(0.63-1.55)	(1.02-2.53)	(0.50-1.25)
30-34	0.28	0.72	0.21	0.74	0.75	0.60	1.02	1.47	0.82
	(0.20-0.39)	(0.52-0.99)	(0.15-0.29)	(0.53-1.02)	(0.54-1.04)	(0.44-0.83)	(0.74-1.41)	(1.06-2.03)	(0.59-1.12)
35-39	0.35	0.78	0.29	0.99	0.94	0.86	1.34	1.72	1.15
	(0.28-0.44)	(0.62-0.99)	(0.23-0.37)	(0.78-1.26)	(0.74-1.19)	(0.68-1.09)	(1.06-1.70)	(1.36-2.18)	(0.91-1.45)
40-44	0.36	0.75	0.27	1.14	1.16	0.88	1.49	1.90	1.15
	(0.29-0.45)	(0.60-0.93)	(0.21-0.34)	(0.91-1.42)	(0.92-1.45)	(0.70-1.10)	(1.19-1.87)	(1.52-2.38)	(0.92-1.44)
45-49	0.31	0.63	0.21	0.94	0.99	0.70	1.25	1.63	0.91
	(0.24-0.39)	(0.50-0.80)	(0.17-0.27)	(0.74-1.19)	(0.78-1.25)	(0.55-0.88)	(0.99-1.58)	(1.28-2.05)	(0.72-1.15)
50-54	0.24	0.52	0.16	0.71	0.83	0.52	0.95	1.36	0.68
	(0.19-0.30)	(0.42-0.65)	(0.12-0.20)	(0.57-0.89)	(0.66-1.04)	(0.42-0.65)	(0.76-1.19)	(1.08-1.70)	(0.54-0.85)
55-59	0.22	0.47	0.15	0.77	0.85	0.59	0.99	1.32	0.74
	(0.18-0.27)	(0.38-0.59)	(0.12-0.19)	(0.62-0.96)	(0.69-1.05)	(0.48-0.73)	(0.80-1.23)	(1.07-1.64)	(0.60-0.92)
60-64	0.15	0.32	0.11	0.58	0.61	0.45	0.73	0.93	0.55
	(0.12-0.19)	(0.26-0.39)	(0.09-0.13)	(0.47-0.71)	(0.49-0.75)	(0.36-0.55)	(0.59-0.90)	(0.75-1.14)	(0.45-0.68)
65-69	0.12	0.24	0.09	0.45	0.47	0.40	0.57	0.71	0.49
	(0.10-0.15)	(0.19-0.29)	(0.07-0.11)	(0.37-0.55)	(0.38-0.58)	(0.32-0.49)	(0.46-0.70)	(0.58-0.87)	(0.40-0.60)
70-74	0.13	0.25	0.10	0.43	0.47	0.37	0.56	0.72	0.47
	(0.10-0.15)	(0.20-0.31)	(0.08-0.12)	(0.35-0.54)	(0.38-0.58)	(0.30-0.46)	(0.45-0.69)	(0.58-0.89)	(0.38-0.58)
75-79	0.11	0.25	0.08	0.40	0.48	0.31	0.51	0.72	0.39
	(0.09-0.13)	(0.20-0.31)	(0.06-0.10)	(0.32-0.50)	(0.38-0.59)	(0.25-0.38)	(0.41-0.63)	(0.58-0.89)	(0.31-0.48)
80-84	0.07	0.18	0.05	0.29	0.38	0.22	0.36	0.56	0.27
	(0.06-0.09)	(0.15-0.23)	(0.04-0.06)	(0.24-0.36)	(0.30-0.46)	(0.18-0.27)	(0.29-0.45)	(0.45-0.69)	(0.22-0.34)
85-89	0.04	0.11	0.03	0.17	0.23	0.12	0.21	0.34	0.15
	(0.03-0.05)	(0.09-0.14)	(0.02-0.03)	(0.14-0.22)	(0.18-0.29)	(0.10-0.16)	(0.16-0.26)	(0.27-0.43)	(0.12-0.19)
≥90	0.02	0.06	0.02	0.08	0.12	0.07	0.10	0.18	0.09
	(0.01-0.03)	(0.04-0.08)	(0.01-0.02)	(0.06-0.11)	(0.09-0.16)	(0.05-0.09)	(0.08-0.14)	(0.14-0.24)	(0.06-0.11)
Total	2.65	6.09	1.97	8.43	9.09	6.68	11.08	15.18	8.65
	(2.05-3.43)	(4.67-7.95)	(1.52-2.56)	(6.56-10.85)	(7.07-11.71)	(5.19-8.60)	(8.61-14.28)	(11.74-19.67)	(6.71-11.16)

PAD – peripheral artery disease, CI – confidence interval

## DISCUSSION

In this study, we conducted a systematic review and meta-analysis of all published evidence on PAD prevalence based on ABI measurement in China. By applying comprehensive search strategies and strict eligibility criteria, the model-based estimates of PAD prevalence across different age groups and between genders were presented. In the present study, the prevalence of PAD increased with age in both males and females, and the increasing rates started to be most pronounced from 60 years. The gender difference of PAD prevalence in Chinese population was assessed, with females at a higher risk of developing PAD than males. Furthermore, three major risk factors for PAD were also confirmed, which were current smoking, hypertension and diabetes. Our study also provided quantitative estimates of the magnitude of affected population in China. Between 2000 and 2020, the number of people with PAD is expected to increase by almost 40%, from 29.44 million (95% CI = 22.51-38.62) to 41.13 million (95% CI = 32.00-52.95). In 2010, most PAD cases resided in the rural East region. Collectively, our findings highlight the existence of a considerable burden of PAD in the general Chinese population.

To the best of our knowledge, our review provided the first national-scale systematic analysis of the prevalence of PAD and its risk factors in LMIC. Pooling data from 132368 participants enrolled in 37 studies, this study included substantially more information than any previous meta-analyses of PAD prevalence in the world. Although previous epidemiological studies have already demonstrated the prevalence of PAD and risk factors in Chinese population, their results varied in many aspects of study characteristics - for example, the entry criteria, diversity of the population with respect to age structure, gender distribution and geography [19,40]. By taking the overall sample and outcome events from the current evidence, our study distinguishes itself from those investigations by providing a pooled nation-wide estimate of PAD prevalence. The sufficient information ensured an adequate power to conduct a reliable assessment and generalise our results in the general population. Another strength of this study is that it adopted comprehensive search strategies in both Chinese and English databases, a dual review process and strict eligibility criteria, thereby increasing its potential to capture all relevant studies. Of note, PAD cases in our included studies were all ascertained by using the cut-off point of ABI lower than 0.90 or equal to or lower than 0.90. This standardised and widely accepted diagnostic criterion has been adopted in our previous estimates of global PAD prevalence and many other epidemiological investigations, so that the comparison between studies and over time can be reliable [4,7,16]. When assessing the risk factors for PAD, we ensured that the definitions of risk factors were similar across all included studies, as well as in the China NCD surveillance system [39]. The multivariate method of risk factor assessment was also consistent across all included studies.

Irrespective of all the merits, it is important to bear the limitations of this study in mind when interpreting the findings. Our targeted population was general Chinese population. Although the effects of different risk factors were also assessed in the present study, our estimates of PAD prevalence and the number of PAD cases cannot be extrapolated to any specific patient populations, for example, diabetic patients. In the assessment of potential risk factors, not all the included studies had retained data on risk factors. Because of the inconsistency of risk factor definitions across the included studies and the paucity of reported ORs, we were only able to explore the effects of nine risk factors, among which four (female gender, current smoking, hypertension and diabetes) were detected as significant risk factors for PAD. However, although no definitive proof of the harm of current drinking, SBP, DBP and BMI was noted in our analysis, we cannot exclude the possibility that these factors might be associated with the occurrence of PAD. Similarly, our analysis of risk factors for PAD does not allow for any certain conclusions about factors for which inadequate data were presented. As an example, hypercholesterolaemia (HC) has been indicated as a significant risk factor in our previous global PAD study, but no relation between HC and PAD can be established in the present study because of insufficient data [7]. Further epidemiological studies on PAD should assess whether these factors are associated with the occurrence of PAD and their magnitudes. In the distribution of national PAD cases, great efforts have been made in our study to explore the distribution of PAD cases in both urban and rural areas of the three Chinese regions. However, this analysis was based on the assumption that the distribution of PAD cases in China was only driven by age structure, gender ratio, and exposures to smoking, diabetes and hypertension, which is obviously far from realistic. Furthermore, the restricted availability of primary data in the included studies limited our ability to generate provincial estimates of PAD prevalence. More epidemiological studies are still largely required.

In our analysis, the prevalence of PAD was found to be age-driven, which is in line with the findings in earlier population-based studies and synthesized evidence [1,7,9]. When compared with the global PAD study, our estimates of PAD prevalence for males in China were higher than those in LMIC before 50 years. The estimates for females in China were lower than those in LMIC across most age groups in adulthood. No previously published meta-analysis has compared the prevalence of PAD in Chinese and other ethnic groups directly. However, an investigation of ethnic-specific PAD prevalence in America might provide a clue, where relatively lower rates were witnessed in Asian American, especially when compared to African American and Hispanics [41].

Furthermore, in our study, PAD was found to be uncommon before the age of 60 years. The prevalence rates then rose sharply with age, such that by the age of above 90 years, more than one in five persons over 90 years of age suffered from PAD. This rather slow increasing rate of PAD prevalence in middle-aged age groups has been observed in Asian American and other ethnic groups in the United States [1,6,41]. Similarly, PAD prevalence was also found to only significantly increase with age after 60 years in Spanish [42]. One possibility is that cardiovascular diseases and their risk factors develop at different rates in different ethnic groups, but all start to accumulate in the older stage. However, this speculation still needs further confirmation by comparing the increasing speed of PAD prevalence by age in different countries. Certain conclusions of this relationship in the Chinese population will also need more systematic and standardised surveys of PAD, where age-specific prevalence should be routinely provided.

The gender difference in PAD prevalence was also noticed in our study, where PAD disproportionately affects females in comparison to males. This finding is in line with the global PAD study, where women in LMIC were more like to have PAD than men. Regarding the higher prevalence of exposure to main risk factors in Chinese males than in Chinese females, especially that of current smoking, the predominantly higher prevalence of PAD in females is somewhat in contrast with our expectation as well as the public’s perception [39]. Several potential mechanisms may exist to explain the inherently higher prevalence of PAD in females, as previously confirmed in other LMIC [7]. These mechanisms need further investigations, as many biological and environmental factors, such as hormonal influence and poverty, may contribute to some extent [16].

Between 2000 and 2020, the number of people with PAD will increase by 40% in China, with around 41 million Chinese adults being projected to be affected by PAD in 2020. Many factors, such as older age structure and an increase in rates of NCDs, might have contributed to this rise [23,43]. Even if risk factors remain stable, it seems that the size of people with PAD will be larger in the coming decades, with ageing of the Chinese population. Despite the fact that PAD represents a large and increasing burden and threat for the health care system and society, it remains an under-recognized issue [14,44]. In addition to the need for more comprehensive assessment of prevalence across the whole nation, more attention should also be given to the planning and implementation of preventative strategies and clinical services.

Another key insight from our analysis is that current smoking, diabetes and hypertension were independently associated with increased risk of PAD in the Chinese population. In the previous global PAD study, the effects of these three risk factors in both HIC and LMIC settings have been confirmed [7]. Although the causality of this relationship cannot be fully addressed because all of the included studies were cross-sectional, our findings still shed some light on the potential benefits of smoking cessation and control of blood glucose and blood pressure. Regular assessment of ABI in patients deemed to be of sufficient absolute risk is also essential for early detection of PAD, and thus should be a routine part in clinical settings. Additional research is also needed to increase our understanding of risk factors for PAD in the Chinese population.

By taking the prevalence of major risk factors in both rural and urban settings of the three Chinese regions, we were able to quantify the number of people with PAD by geography. In our study, more people with PAD were found in rural areas than in urban areas, with rural East encountering most cases. This uneven distribution of PAD cases between urban and rural settings suggests a combined effect of uneven demography and exposure to major risk factors. The relatively younger age structure of the urban settings, especially the urban East, was also implied by our findings.

## CONCLUSIONS

To conclude, the results from our study have shown that PAD is a prevalent disease and major public health challenge in China. PAD is more frequent in elderly and females, current smokers, hypertensives and diabetics in the Chinese population. With rapid ageing trend and emerging NCDs in China, the number of people with PAD will increase by 40% between 2000 and 2020. In 2010, the most PAD cases were in the rural East region. More research and optimal interventions on PAD are required to better identify effective strategies for preventing and treating PAD in China.

## Additional Material

Online Supplementary Document
