# Supplementary material for: National and subnational estimation of the prevalence of peripheral artery disease (PAD) in China: a systematic review and meta-analysis
Source: J Glob Health. 2019 Mar 15;9(1):010601. doi: 10.7189/jogh.09.010601 (PMC6377796; doi:10.7189/jogh.09.010601)
Supplement: Online Supplementary Document [file jogh-09-010601-s001.pdf]

Song et al. J Glob Health Res 2019; 9: 010701

Table S1. Search strategies in six bibliographic databases

Table S2. The year of publication and investigation and corresponding time-lag in the 37 included studies

Table S3. Univariable meta-regression models of factors related to the prevalence of PAD

Table S4. Prevalence of the three major risk factors, by gender, setting and region

Table S5. Characteristics of the 37 included studies

Table S6. Meta-analyses of risk factors for PAD

Table S1. Search strategies in six bibliographic databases

Database	Access date	Subject category	Sub-database	Search terms	Publication date	Search method
CNKI	03/03/2017	Medicine & Public Health	Journal, Featured journal, Doctoral dissertation, Master dissertation, Domestic conferences, International conferences	(SU % '外周动脉疾病' + '外周动脉病'+ '外周动脉病变'+ '外周动脉硬化'+ '下肢动脉疾病'+ '下肢动脉病'+ '下肢动脉病变'+ '下肢动脉硬化'+ '周围动脉疾病'+ '周围动脉硬化'+ ' peripheral artery disease ') AND (SU % '发病率' + '发生率' + '患病率'+ '罹患率' + '现患率'+ '死亡率' + '病死率'+ '流行' + '负担'+ '现况调查'+ '现况研究')	01/01/1990 - 03/03/2017	Comprehensive search: title, keywords and abstract
Wanfang	03/03/2017	Not applicable	Journal articles, Dissertations	(主题:(外周动脉疾病) + 主题:(外周动脉病) + 主题:(外周动脉病变) + 主题:(外周动脉硬化) + 主题:(下肢动脉疾病) + 主题:(下肢动脉病) + 主题:(下肢动脉病变) + 主题:(下肢动脉硬化) + 主题:(周围动脉疾病) + 主题:(周围动脉硬化) + 主题:(peripheral artery disease)) * (主题:(发病率) + 主题:(发生率) + 主题:(患病率) +主题:(罹患率) + 主题:(现患率) + 主题:(死亡率) +主题:(病死率) + 主题:(流行) + 主题:(负担)+ 主题:(现况调查) + 主题:(现况研究))	1990-2017	Comprehensive search: title, keywords and abstract
SinoMed	03/03/2017	Medicine & Public Health	All journals	(外周动脉疾病 or 外周动脉病 or 外周动脉病变 or 外周动脉硬化 or 下肢动脉疾病 or 下肢动脉病 or 下肢动脉病变 or 下肢动脉硬化 or 周围动脉疾病 or 周围动脉硬化 or peripheral artery disease) AND (发病率 or 发生率 or 患病率 or 罹患率 or 现患率 or 死亡率 or 病死率 or 流行 or 负担 or 现况调查 or 现况研究)	1990-2017	Comprehensive search: title, keywords and abstract
PubMed	03/03/2017	Not applicable	Not applicable	((peripheral artery disease or peripheral arterial disease) AND (China OR Chinese) AND (inciden* OR prevalen* OR morbidity OR mortality OR epidemiology)) AND ("1990/01/01"[Date - Publication] : "2017/03/03"[Date - Publication])	01/01/1990 - 03/03/2017	Comprehensive search: all fields
Embase	03/03/2017	Not applicable	Not applicable	1 peripheral artery disease.mp. or exp peripheral occlusive artery disease/	1990-2017	Comprehensive search: all

				2 Chin*.mp.<br>3 incidence/ or inciden*.mp.<br>4 prevalence/ or prevalen*.mp.<br>5 morbidity/<br>6 mortality/<br>7 epidemiology/<br>8 3 or 4 or 5 or 6 or 7<br>9 1 and 2 and 8<br>10 limit 9 to yr="1990 -2017"		fields
Medline	03/03/2017	Not applicable	Not applicable	1 peripheral artery disease.mp. or exp Peripheral Arterial Disease/<br>2 Chin*.mp.<br>3 incidence/ or inciden*.mp.<br>4 prevalence/ or prevalen*.mp.<br>5 morbidity/<br>6 mortality/<br>7 epidemiology/<br>8 3 or 4 or 5 or 6 or 7<br>9 1 and 2 and 8<br>10 limit 9 to yr="1990 -Current"	01/01/1990 - 03/03/2017	Comprehensive search: all fields

Table S2. The year of publication and investigation and corresponding time-lag in the 37 included studies

Author	Year published	Median investigation date	Time-lag (year)
Liu GC et al.	2005	2004	1
Zheng LQ et al.	2006	2005	1
Liu H et al.	2007	2005	2
Wang LL et al.	2007	2006	1
Zhou YY et al.	2009	2008	1
Chen JH et al.	2010	2008	2
Wang Y et al.	2009	2008	1
An W et al.	2010	NA	NA
Zhang X et al.	2010	2009	1
Yang XM et al.	2010	2009	1
Wang GP et al.	2010	2008	2
Xu SQ et al.	2010	2008	2
Zhou L et al.	2012	2010	2
Bai XQ et al.	2012	2008	4
Xie XL et al.	2010	2007	3
Liu L et al.	2013	NA	NA
Zhu HM	2013	NA	NA
Wang LY et al.	2013	2010	3
Liu YP et al.	2014	2011	3
Chuang SY et.al	2005	2002	3
Yao H et.al	2006	2001	5
J.Woo et.al	2012	2001	11
J.Woo et.al	2006	2006	0
Samuel Y.S.Wong et.al	2007	2002	5
Samuel Y.S.Wong et.al	2009	NA	NA
Xiang Y et.al	2011	2010	1
Chen P	2012	2009	3
Hu BC et.al	2013	2006	7
Liang YJ et.al	2014	2011	3
Yang JH	2014	2010	4
Lin LM et.al	2014	2009	5

Pan XH et.al	2015	NA	NA
Han HF et.al	2014	2011	3
Lin CH et.al	2015	2009	6
Wen JP et.al	2015	2007	8
Wang AX et.al	2016	2011	5
Huang S	2016	NA	NA

Based on the information from 31 studies, the average time lag between the year of publication and the year of investigation was 3.09.

Table S3. Univariable meta-regression models of factors related to the prevalence of PAD

Moderator	Number of studies	Number of data points	β	95 % CI	P value
Intercept	37	166	-2.710	[-2.912]-[-2.508]	<0.001
Age	37	166	0.024	0.021-0.027	<0.001
Gender-Male	33	154	-0.299	[-0.351]-[-0.246]	<0.001
Setting-Rural	30	128	-0.054	[-0.509]- 0.401	0.816
Investigation year	37	166	0.018	[-0.014]- 0.049	0.271

Note: coefficients indicate log odds ratios (ORs).

In the univariable meta-regression analysis, age and gender were significantly associated with PAD prevalence, whereas no setting difference or secular trend was found in the distribution of PAD prevalence. Therefore, the final multivariate meta-regression model was used to produce the age- and gender-specific prevalence estimates.

Table S4. Prevalence of the three major risk factors, by gender, setting and region

Region	Male			Female		
**Urban**						
	**Current smoking**	**Hypertension**	**Diabetes**	**Current smoking**	**Hypertension**	**Diabetes**
Central	51.8%	37.0%	11.8%	3.2%	31.2%	9.0%
East	50.2%	41.2%	15.2%	2.5%	33.7%	12.9%
West	57.9%	30.8%	11.1%	2.7%	28.2%	10.2%
**Rural**						
	**Current smoking**	**Hypertension**	**Diabetes**	**Current smoking**	**Hypertension**	**Diabetes**
Central	53.3%	34.7%	10.0%	3.0%	33.4%	8.8%
East	54.6%	37.7%	9.5%	2.1%	33.0%	8.9%
West	52.9%	28.6%	6.7%	1.8%	28.6%	6.0%

The prevalence of current smoking, hypertension and diabetes was derived from the 2010 China Non-communicable and Chronic Disease surveillance, which included 162 surveillance points across the 31 provinces in China. Hypertension was defined as an SBP ≥140mmHg and(or) DBP ≥90mmHg or being previously diagnosed. Diabetes was defined as fasting blood-glucose ≥7.0mmol/L and (or) 2h oral glucose tolerance test ≥11.1mmol/L or being previously diagnosed.

Table S5. Characteristics of the 37 included studies

Author	Year Published	Province	Setting	Study year	Sampling	Age range	Sample size	Cases	Prevalence (%)	Title
Liu GC et al.	2005	Zhejiang	Rural	2004	Cluster sampling	35-89	2668	57	2.14	浙江省舟山渔区外周动脉病患病率调查
Zheng LQ et al.	2006	Sichuan	Rural	2005	Random cluster sampling	18-39	590	60	10.17	四川盐边县不同体质指数人群的踝臂指数及下肢外周动脉病患病率调查
Liu H et al.	2007	Sichuan	Rural	2005	NA	40+	643	64	9.95	高敏感 C 反应蛋白与外周动脉疾病的关系
Wang LL et al.	2007	Shanghai	Urban	2006	Custer sampling	60-97	2360	205	8.69	上海市程桥社区老年人外周动脉疾病患病率的调查
Zhou YY et al.	2009	Guangdong	Rural	2008	Random cluster sampling	60-93	1447	165	11.40	1447 例容桂社区老年人下肢动脉疾病的流行病学调查
Chen JH et al.	2010	Guangdong	Rural	2008	Random cluster sampling	60-93	232	40	17.24	老年人群下肢动脉疾病调查及社区护理干预
Wang Y et al.	2009	Beijing, Shanghai, Changsha, Guangdong, Inner Mongolia, Xinjiang	Urban	2008	Stratified random cluster sampling	18+	21152	652	3.08	中国自然人群下肢外周动脉疾病患病率及相关危险因素
An W et al.	2010	NA (Nine areas)	Mixed	2007	Random cluster sampling	35+	16511	1024	6.20	高血压与外周动脉疾病的关系
Zhang X et al.	2010	Beijing	Urban	2009	Random sampling	60-94	988	127	12.85	踝臂指数评价老年外周动脉疾病的价值
Yang XM et al.	2010	Inner Mongolia	Mixed	2009	Regular health examination	40-89	1040	138	13.27	中老年人群踝臂指数减低的相关因素研究
Wang GP et al.	2010	Xinjiang	Urban	2008	Random cluster sampling	60-93	1046	255	24.38	社区老年人下肢动脉硬化闭塞症患病率调查及相关危险因素分许
Xu SQ et al.	2010	Zhejiang	Urban	2008	Regular health examination	26-81	2994	73	2.44	2994 名职工踝臂指数调查
Zhou L et al.	2012	Shanghai	Urban	2010	Cluster sampling	40+	5435	386	7.10	社区人群中外周动脉病与体质量指数相关性的研究
Bai XQ et al.	2012	Beijing	Urban	2008	Random sampling	20-96	7819	136	1.74	代谢综合征及其组分对踝臂指数的影响
Xie XL et al.	2010	Shanghai/Inner Mongolia	Mixed	2007	Stratified random cluster sampling	15.3-93.5	2569	80	3.11	上海与内蒙古自治区两地自然人群踝臂指数的调查研究
Liu L et al.	2013	Guizhou	Mixed	2010	Random cluster sampling	40+	4333	149	3.44	贵阳市 40 岁及以上自然人群代谢综合征并外周动脉疾病的患病率调查

Zhu HM	2013	Shanghai	Rural	2010	Custer sampling	65+	1492	51	3.42	上海社区老年人群踝臂脉搏波传导速度与踝臂指数的调查研究
Wang LY et al.	2013	Jiangsu	Urban	2010	Custer sampling	35+	1022	47	4.60	社区人群下肢外周血管病流行病学调查
Liu YP et al.	2014	Sichuan	Mixed	2011	Random sampling	45+	6563	128	1.95	中老年健康体检者下肢外周动脉疾病患病情况及危险因素分析
Chuang SY et.al	2005	Taiwan	Rural	2002	Cluster sampling	40-79	1329	29	2.18	Combined use of brachial-ankle pulse wave velocity and ankle-brachial index for fast assessment of arteriosclerosis and atherosclerosis in a community
Yao H et.al	2006	Beijing	Urban	2001	Stratified sampling	60+	2334	198	8.48	Prevalence of peripheral arterial disease and its association with smoking in a population-based study in Beijing, China
J.Woo et.al	2012	Hongkong	Urban	2001	Stratified sampling	65+	3798	262	6.90	THE ALU POLYMORPHISM OF ANGIOTENSIN I CONVERTING ENZYME (ACE) AND ATHEROSCLEROSIS, INCIDENT CHRONIC DISEASES AND MORTALITY IN AN ELDERLY CHINESE POPULATION
J.Woo et.al	2006	Hongkong	Urban	2006	Stratified sampling	65+	3998	274	6.85	Correlates for a low ankle–brachial index in elderly Chinese
Samuel Y.S.Wong et.al	2007	Hongkong	Urban	2002	Convenience stratified sampling	65+	3985	272	6.83	Clinically relevant depressive symptoms and peripheral arterial disease in elderly men and women. Results from a large cohort study in Southern China
Samuel Y.S.Wong et.al	2009	Hongkong	Urban	2006	Stratified sampling	65+	1561	95	6.09	Sexual Activity, Erectile Dysfunction and Their Correlates among 1,566 Older Chinese Men in Southern China
Xiang Y et.al	2011	Xinjiang	Rural	2010	Stratified random cluster sampling	35-84	2082	97	4.66	新疆阿勒泰福海地区哈萨克族居民 PAD 患病率及危险因素分析
Chen P	2012	Xinjiang	Mixed	2009	Stratified random cluster sampling	35-84	8389	542	6.46	新疆维吾尔族、哈萨克族外动脉疾病患病率及危险因素分析
Hu BC et.al	2013	Shanghai	Rural	2006	Cluster sampling	NA	951	31	3.26	Ankleebrachial index in relation to the natriuretic peptide system polymorphisms and urinary sodium excretion in Chinese
Liang YJ et.al	2014	Shandong	Rural	2011	Cluster sampling	NA	1499	85	5.67	Cardiovascular Risk Factor Profiles for Peripheral Artery Disease and Carotid Atherosclerosis among Chinese Older People: A Population-Based Study
Yang JH	2014	Beijing	Urban	2010	Stratified random cluster sampling	60+	2101	147	7.00	老年人代谢性疾病及周围动脉闭塞性疾病基因关联研究
Lin LM et.al	2014	Hebei	Urban	2009	Stratified random sampling	40+	5184	182	3.51	空腹血糖与踝臂指数的相关性
Pan XH et.al	2015	Zhejiang	Mixed	2012	Stratified cluster samp ling	30-74	1730	234	13.53	居民踝臂指数水平调查
Han HF et.al	2014	Hebei	Urban	2011	Regular health examination	NA	2408	113	4.69	不同性别人群踝臂指数的分布情况

Lin CH et.al	2015	Taiwan	Urban	2009	Cluster sampling	65+	1036	74	7.14	Association between frailty and subclinical peripheral vascular disease in a community-dwelling geriatric population: Taichung Community Health Study for Elders
Wen JP et.al	2015	Hebei	Rural	2007	Cluster sampling	30+	4748	212	4.47	Comparisons of Different Metabolic Syndrome Definitions and Associations with Coronary Heart Disease, Stroke, and Peripheral Arterial Disease in a Rural Chinese Population
Wang AX et.al	2016	Hebei	Urban	2011	Stratified random sampling	40+	3048	161	5.28	A low ankle-brachial index is associated with cognitive impairment:The APAC study
Huang S	2016	Guangxi	Rural	2013	Stratified random sampling	NA	1283	43	3.35	永福县和柳江县农村居民踝臂指数及其相关危险因素调查

Table S6. Meta-analyses of risk factors for PAD

Risk factor 1-Advanced age (per year)

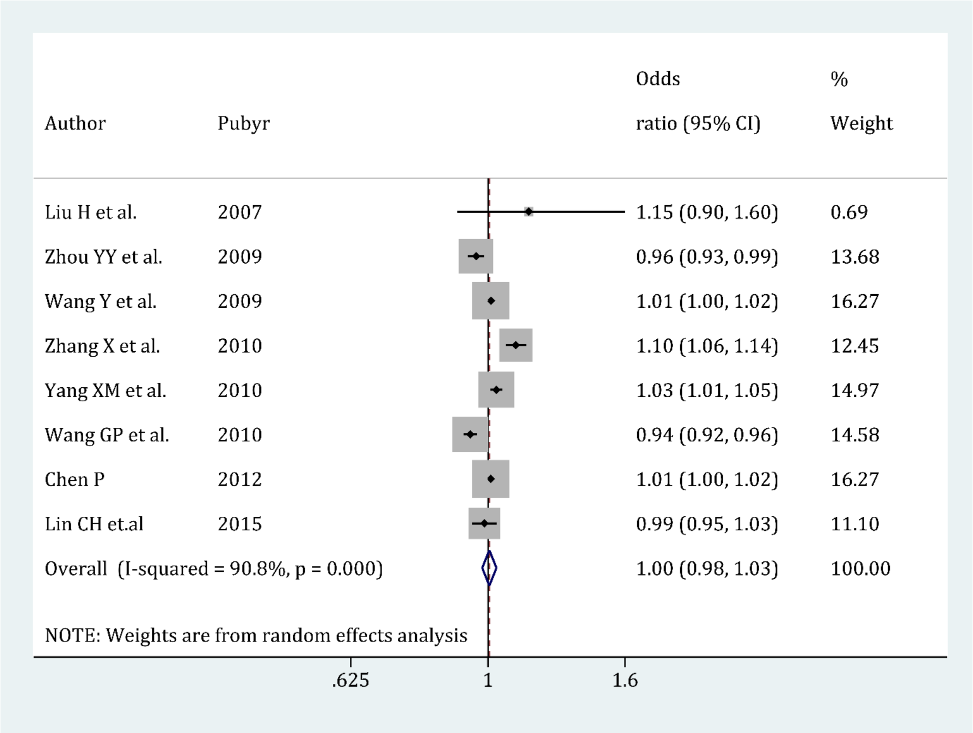

Risk factor 2-Female gender

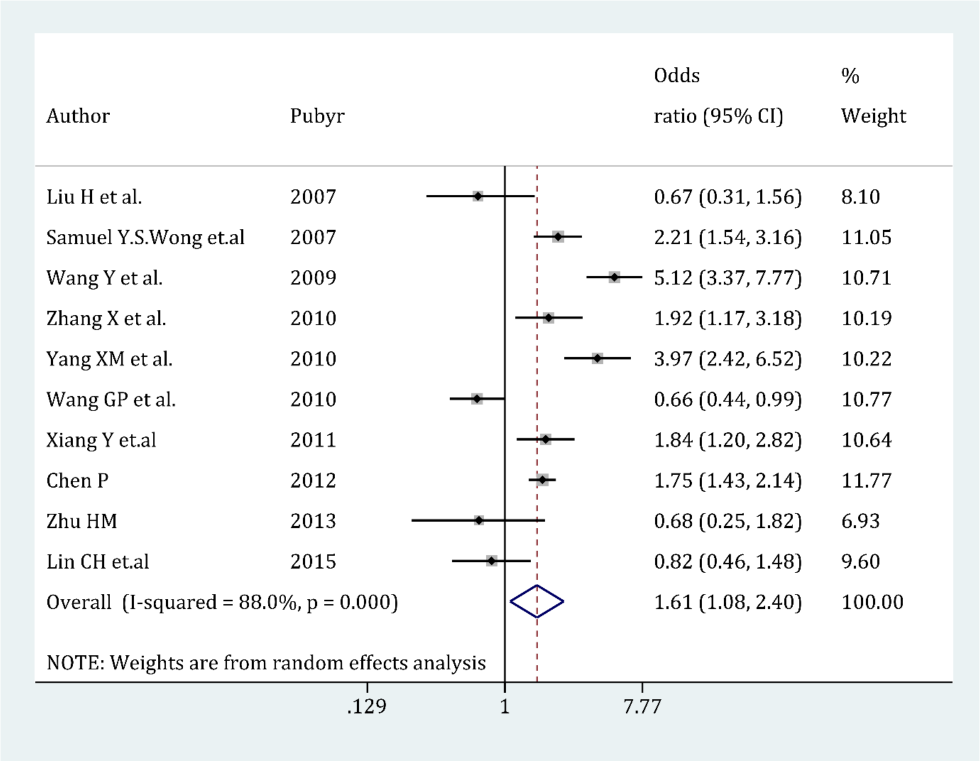

Risk factor 3-Current smoking

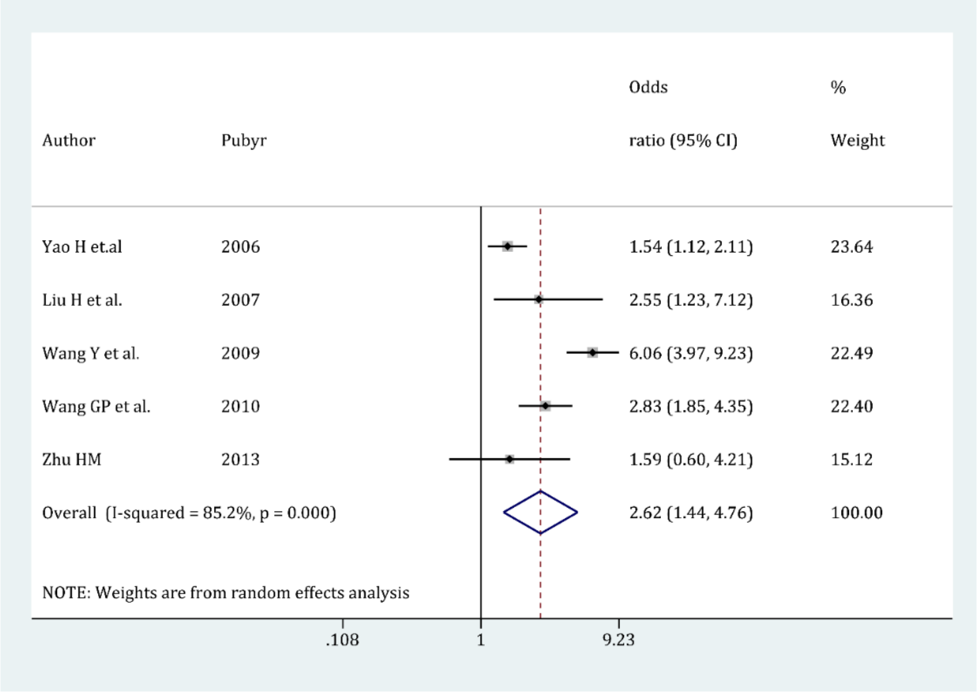

Risk factor 4-SBP (mmHg)

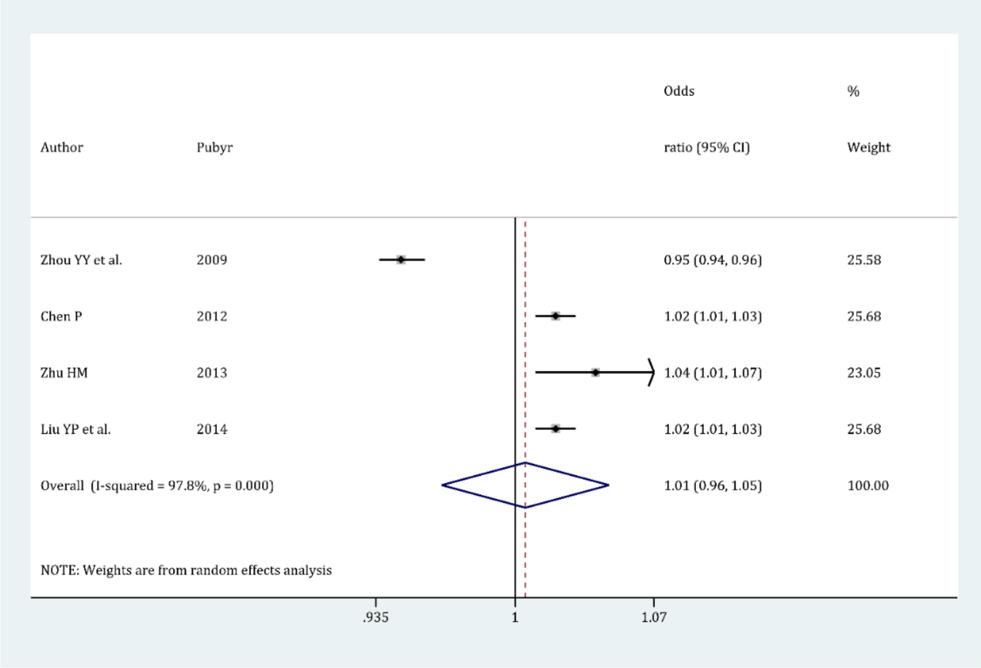

Risk factor 5-DBP (mmHg)

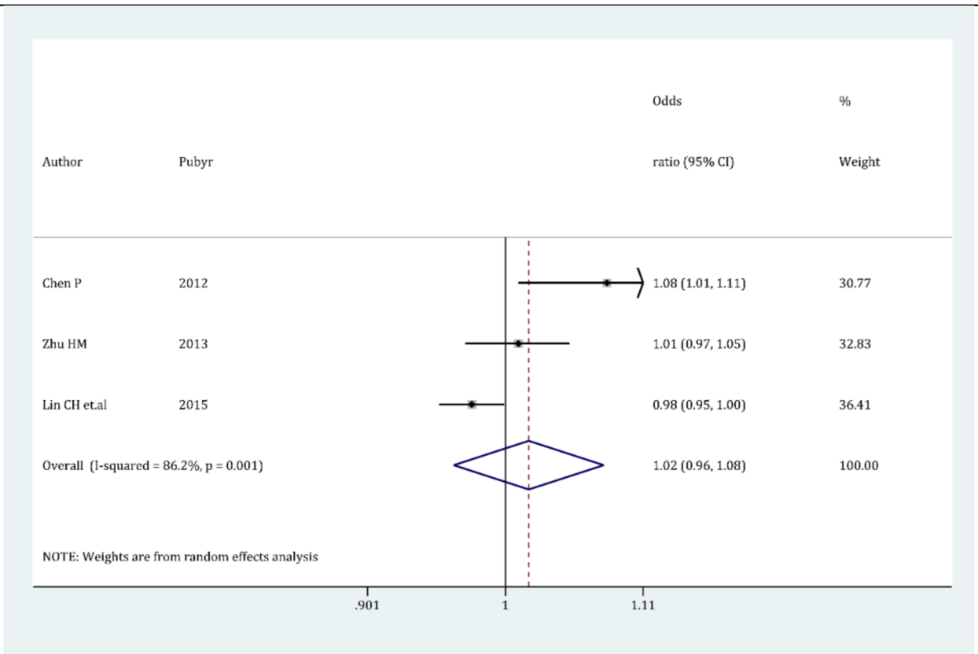

Risk factor 6-Current drinking

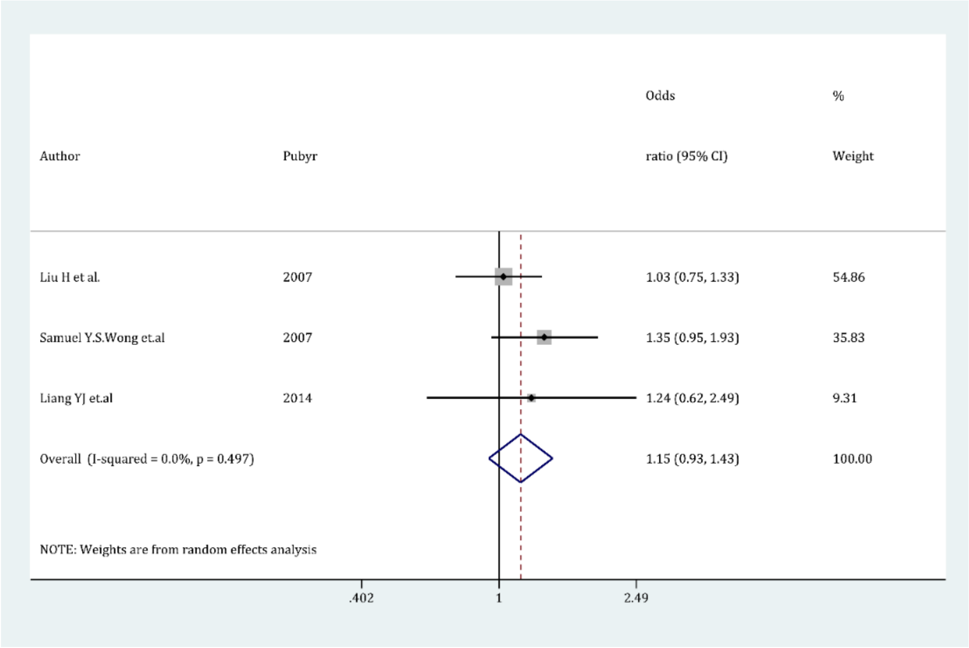

Risk factor 7-BMI (kg/m²)

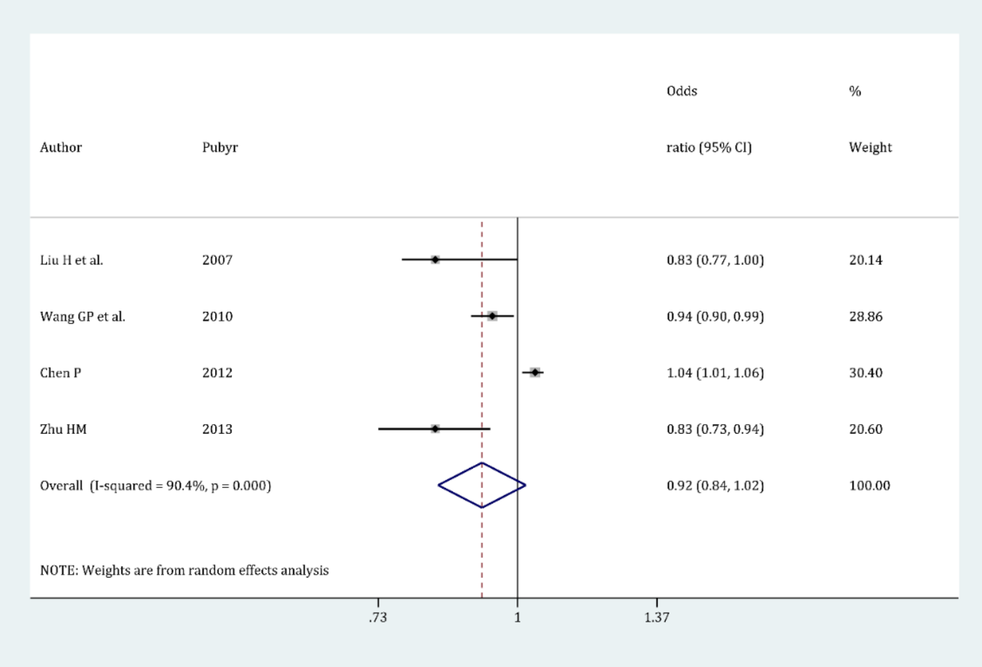

Risk factor 8-Hypertension

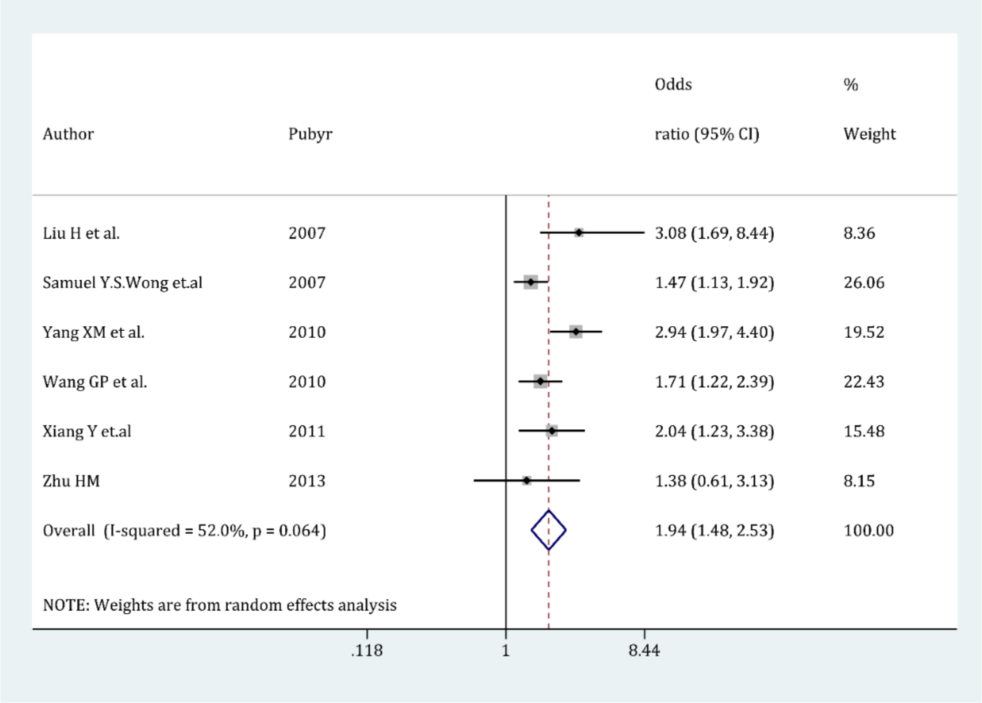

Risk factor 9-Diabetes

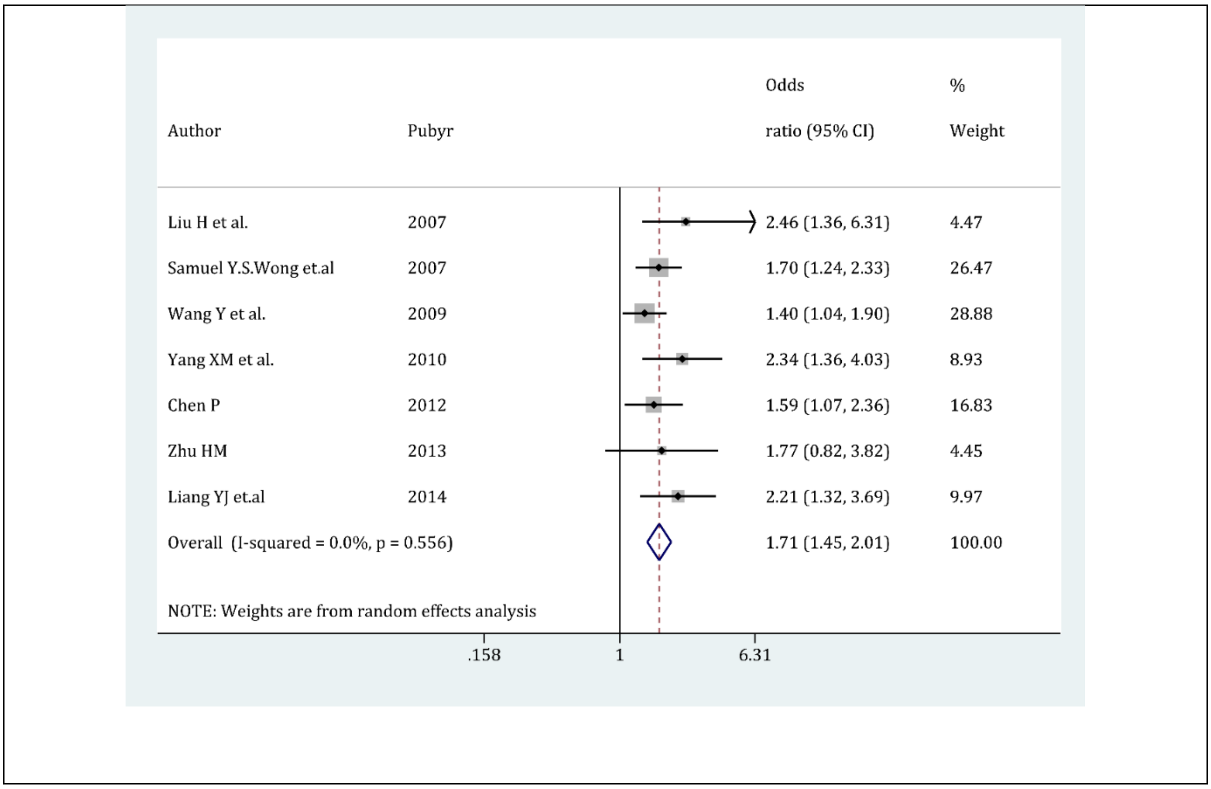

Author
Pubyr
Odds ratio (95% CI)
% Weight
Liu H et al. 2007 2.46 (1.36, 6.31) 4.47
Samuel Y.S.Wong et.al 2007 1.70 (1.24, 2.33) 26.47
Wang Y et al. 2009 1.40 (1.04, 1.90) 28.88
Yang XM et al. 2010 2.34 (1.36, 4.03) 8.93
Chen P 2012 1.59 (1.07, 2.36) 16.83
Zhu HM 2013 1.77 (0.82, 3.82) 4.45
Liang YJ et.al 2014 2.21 (1.32, 3.69) 9.97
Overall (I-squared = 0.0%, p = 0.556) 1.71 (1.45, 2.01) 100.00
NOTE: Weights are from random effects analysis
.158
1
6.31
